# IL-15 enhances cross-reactive antibody recall responses to seasonal H3 influenza viruses *in vitro*

**DOI:** 10.12688/f1000research.12999.1

**Published:** 2017-11-15

**Authors:** Junqiong Huang, Shannon P. Hilchey, Jiong Wang, Jessica Gerigan, Martin S. Zand

**Affiliations:** 1 School of Laboratory Medicine, Zunyi Medical University, Zunyi Guizhou, 563099, China; 2 Division of Nephrology, University of Rochester Medical Center, Rochester , NY, 14642, USA

**Keywords:** Influenza immunity, B Cell, CpG ODN, IL-15, hemagglutinin stalk

## Abstract

**Background: **Recently, several human monoclonal antibodies that target conserved epitopes on the stalk region of influenza hemagglutinin (HA) have shown broad reactivity to influenza A subtypes. Also, vaccination with recombinant chimeric HA or stem fragments from H3 influenza viruses induce broad immune protection in mice and humans. However, it is unclear whether stalk-binding antibodies can be induced in human memory B cells by seasonal H3N2 viruses.

**Methods:** In this study, we recruited 13 donors previously exposed to H3 viruses, the majority (12 of 13) of which had been immunized with seasonal influenza vaccines. We evaluated plasma baseline strain-specific and stalk-reactive anti-HA antibodies and B cell recall responses to inactivated H3N2 A/Victoria/361/2011 virus *in vitro* using a high throughput multiplex (mPlex-Flu) assay.

**Results: **Stalk-reactive IgG was detected in the plasma of 7 of the subjects. Inactivated H3 viral particles rapidly induced clade cross-reactive antibodies in B cell cultures derived from all 13 donors. In addition, H3 stalk-reactive antibodies were detected in culture supernatants from 7 of the 13 donors (53.8%).  H3 stalk-reactive antibodies were also induced by H1 and H7 subtypes. Interestingly, broadly cross-reactive antibody recall responses to H3 strains were also enhanced by stimulating B cells *in vitro *with CpG _2006 _ODN in the presence of IL-15. H3 stalk-reactive antibodies were detected in  CpG _2006_ ODN + IL-15 stimulated B cell cultures derived from 12 of the 13 donors (92.3%), with high levels detected in cultures from 7 of the 13 donors.

**Conclusions:** Our results demonstrate that stalk-reactive antibody recall responses induced by seasonal H3 viruses and CpG _2006_ ODN can be enhanced by IL-15.

## Introduction

Worldwide, annual influenza epidemics are estimated to result in about 3 to 5 million cases of severe illness, and about 250,000 to 500,000 deaths ^1, 2^. Preventive vaccination is the major intervention currently used to prevent influenza infections ^3, 4^, and is designed to elicit IgG antibodies directed against the hemagglutinin viral surface protein (HA). Current vaccine formulations elicit a potent immune response against viruses that are closely matched to the vaccine strain, largely through targeting epitopes on the globular head of HAs of influenza A H1N1 and H3N2 subtypes and influenza B strains. However, antigenic drift of influenza virus, which is caused by an accumulation of point mutations within the HA sequences, frequently occurs in influenza A strains and this is particularly true for H3 influenza A strains ^5– 7^. Individuals who were infected by or vaccinated against H3 influenza viruses circulating in prior years may thus be susceptible to new viral strains.

Since jumping species to humans in 1968, H3N2 swine flu viruses have been responsible for several seasonal pandemics, resulting in both prolonged duration of the influenza season and greater disease severity ^8, 9^. Under the selective pressure of host immunity, H3N2 influenza virus HAs have undergone progressive antigenic drift. This became particularly problematic during the 2014–2015 influenza season, when H3N2 strains became predominant and were antigenically and genetically distinct from the A/Texas/50/2012 (A/Tex12) vaccine strain ^10, 11^. The resulting antigenic mismatch between the vaccine strain and circulating H3 viruses, lead to extremely low vaccine effectiveness in the northern hemisphere ^12^.

In contrast to the variable HA head domain, epitopes within the HA stalk domain are highly conserved and have become a main target for development of novel treatments using either antibody-based vaccine design or passive immunotherapy. Several human monoclonal antibodies that target highly conserved epitopes on the stalk region of influenza HA display broad reactivity with group 1 and/or group 2 viruses and protect against lethal challenge with influenza viruses *in vivo* ^13– 16^. Animals immunized against H3 stalk elicited broadly cross-reactive antibodies, resulting in protection from challenges with viruses that are of the same HA subtype and/or group ^17– 19^. Others have found that vaccination with a divergent hemagglutinin can increase the frequency of B cells encoding broad influenza A-neutralizing antibodies ^20^. However, stalk-reactive antibodies are rarely found in individuals vaccinated with traditional in-activated influenza virus seasonal vaccines ^21– 23^. Recent studies have detected broadly cross-reactive, anti-stalk IgG antibodies in people vaccinated with the pandemic H1N1 A/California/07/09 (pdm A/Cali09) strain ^24, 25^. Recombinant chimeric HA from H3 viruses have also been shown to elicit broad immune protection in mice and humans ^26, 27^. However, it is unclear whether stalk-binding antibodies can be induced in human memory B cells using seasonal H3N2 viruses.

Several strategies have been employed in an attempt to improve broadly cross-reactive IgG production by application of a non-specific stimulus, the most common of which is the addition of various adjuvants to promote increased antibody secretion. In addition, the application of various cytokines has also been studied to increase antibody production, including IL-15. IL-15, a member of the 4- *α*-helix bundle family of cytokines, signals via hetero-trimeric receptors involving the IL-2 receptor *β* chain (IL2R *β* ), a common *γ* chain (IL2R *γ*c), which is also required for signaling by IL-2, IL-4, IL-7, IL-9 and IL-21, and a unique *α* subunit (IL-15R *α*) that confers receptor specificity to IL-15 ^28^. Some cytokines that signal through the common IL-2R *γ* chain have been shown to increase activated naive and memory B cell IgG secretion rates ^29^. IL-15 signals through the activation of JAK2, p38 and ERK1/2 MAPK, SYK kinase and the NF-kB transcriptional factor ^30^. Due to the common *γ*c and *β* chain, IL-15 shares certain functions with IL-2, including T cell proliferation, the generation of cytotoxic T cells, immunoglobulin synthesis by B cells and the generation and persistence of NK cells ^31, 32^. IL-15 has been shown to play an essential role in the proliferation of memory B cells and Ig production *in vivo* ^33^.

In addition to promoting the proliferation, differentiation, and IgG secretion of germinal center B cells, IL-15 is also involved in the generation and maintenance of long-term serologic memory ^29, 34, 35^. IL-15 adjuvant has been reported to increase IgG production in animals immunized with influenza vaccines ^36^, and DIII antigens of Japanese encephalitis virus and West Nile virus ^37^. IL-15 adjuvanted immunization with a DNA vaccine comprised of the N1 and NP genes from the H5N1 influenza virus induced early and high antibody response in chickens ^38^. In addition, IL-15 participates in the homing of immature B cells and maintenance of the B cell repertoire ^39^. Finally, IL-15 signaling appears to be essential to CD4 T cell and B cell activation by CpG ODN signaling through TLR9 ^40^, suggesting further synergy between existing vaccine adjuvants and IL-15.

As a vaccine adjuvant, IL-15 has been used for the HIV vaccine and cancer trails ( www.clinicaltrials.gov: NCT00775424, NCT00115960, NCT00528489 and NCT01021059). Previous studies have shown that IL-15 promotes the survival, proliferation and Ig production of memory B cells ^41, 42^. In the current study, we examine human memory B cell IgG recall responses to H3N2 influenza virus in the presence of CpG _2006_ ODN activation with IL-15 co-stimulation *in vitro*. Our results demonstrate that stalk-reactive IgG antibodies induced by B cell exposure to H3 viruses *in vitro*, in the presence of CpG _2006_ ODN, are enhanced by IL-15 co-administration. In addition, IgG antibodies elicited by H3 viruses and/or IL-15 broadly bound to influenza HAs from both group 1 and group 2 influenza strains, which suggests potential use of CpG adjuvants and/or IL-15 agonists in influenza vaccination strategies.

## Methods

### Study subjects

This study was approved by the Institutional Review Board at the University of Rochester Medical Center (RSRB protocol RSRB00066522). Subjects were recruited at the University of Rochester through local advertisement, and signed a written statement of informed consent prior to phlebotomy for the study. A total of 13 adults with an age range of 26 to 63 years (mean 43.7 years) were included in the study. Twelve study subjects (S1–S3, S5–S13) gave a history of being previously vaccinated with seasonal influenza vaccines, while one subject (S4) indicated that they had never received any influenza vaccine. Peripheral blood was obtained from all subjects as part of the study for B cell stimulation and analysis of baseline influenza-specific antibodies.

### mPlex-Flu assay

The levels of HA-reactive IgG were measured in plasma and *in vitro* stimulated B cell culture supernatants using the mPlex-Flu assay, as previously described ^43^. The assay panels included whole HA or the head segments of influenza group 1, group 2, B strain and chimeric HA, as listed in Table 1. Briefly, 25 *µ*L of plasma dilution (1:5000) or undiluted culture supernatants were incubated with 25 *µ*L of a panel of beads coupled with HAs at room temperature for two hours on a rotary shaker (500 rpm) in the dark. Then 150 *µ*L of phycoerythrin (PE) conjugated goat anti-human IgG (Southern Biotech, Birmingham, Al) was added and incubated at room temperature for 2 hours on a rotary shaker (500 rpm) in the dark. After wells were washed twice with PBS (pH 7.2) containing 0.1% BSA (MP Biomedical, LLC, France) and 0.1% Brij-35 (Thermo Scientific, Waltham, MA), IgG levels were analyzed on Magpix Multiplex Reader (Luminex, Austin, TX). All samples were measured in duplicate.

**Table 1.** mPLEX-Flu Hemagglutinin Panel.

Strain	Gene Bank Accession	Full Strain Name	Abbreviation
H1N1	AF117241.1	A/South Carolina/1/18	A/SC18
H1N1	CY148243.1	A/Puerto Rico/8/1934	A/PR8
H1N1	DQ508897.1	A/USSR/90/1977	A/USSR77
H1N1	DQ508889.1	A/Texas/36/1991	A/Tex91
H1N1	CY125100.1	A/New Caledonia/20/1999	A/NewCal99
H1N1	FJ966974.1	A/California/07/2009	pdm A/Cali09 ^†^
H2N2	L20407.1	A/Japan/305-/1957	A/Jap57
H3N2	CY112249.1	A/Hong Kong/1/1968	A/HK68
H3N2	CY009348.1	A/Port Chalmers/1/1973	A/PC73
H3N2	M57630.1	A/Alabama/1/1981	A/Ala81
H3N2	GQ293081.1	A/Perth/16/2009	A/Perth09
H3N2	DQ508865	A/Panama/2007/1999	A/Pan99
H3N2	KM821347	A/Victoria/361/2011	A/Vic11 ^†^
H3N2	KC892248.1	A/Texas/50/2012	A/Tex12
H3N1	EPI_ISL_164719Ê ^‡^	A/Switzerland/9715293/2013	A/Swi13
H5N1	EF541403.1	A/Viet Nam/1203/2004	A/Viet04
H6N1	KJ162860.1	A/chicken/Taiwan/67/2013	A/TW13
H7N1	EF470586	A/rhea/North Carolina/39482/1993	A/rheaNC93
H7N1	KF695239	A/mallard/Netherlands/12/2000	A/malNeth00
H7N1	KF021597	A/Shanghai/1/2013	A/SH13 ^†^
H9N2	AY206676.1	A/guinea fowl/Hong Kong/WF10/1999	A/gfHK99
H9N2	ADC41843.1	A/Hong Kong/33982/2009	A/HK09 *
B	CY115343	B/Brisbane/60/2008	B/Bris08
B	KF752446.1	B/Massachusetts/2/2012	B/Mass12
B	EPI_ISL_165882 ^‡^	B/Phuket/3027/2013	B/Phu13
H5	Head	Head (C52-C277) of A/Indonesia/5/05	H5 head
cH5/3	Chimera	Head of A/Indonesia/5/05, stalk of A/A/Perth/16/2009	cH5/3
cH5/1	Chimera	Head of A/Indonesia/5/05, stalk of A/California/07/2009	cH5/1Cal09
cH5/1	Chimera	Head of A/Indonesia/5/05, stalk of A/Puerto Rico/8/1934	cH5/1PR8
cH4/7	Chimera	Head of A/duck/Czech/1956(H4), stalk of A/Shanghai/1/2013	cH4/7
H9	Head	Head (C52-C277) of A/guinea fowl/ Hong Kong/WF10/1999	H9 head

*BPL inactivated virus used for *in vitro* stimulation only ^†^ Recombinant HA used in mPlex-Flu and BPL inactivated virus for *in vitro* stimulation ^‡^GISAID accession number

### *In vitro* activation of memory B cells

Primary human B cells were isolated and activated with CpG _2006_ ODN, as previously described ^29, 44^. Cells were negatively enriched from peripheral blood by treatment with an EasySep Human B Cell Enrichment Kit (STEMCELL Technologies, Cambridge, MA), followed by magnetic separation according to the manufacturer’s instructions. B cells were resuspended in complete medium (RPMI 1640 supplemented with 10% heat-inactivated fetal bovine serum, 100 units/mL penicillin G, and 100 *µ*g/mL streptomycin) and were cultured (5 *x*10 ^5^/well, 1 mL/ well) with 6 *µ*g/mL CpG _2006_ (Integrated DNA Technologies, San Diego, CA) alone or together with 10 *µ*g/mL of BPL inactivated A/Victoria/361/2011 (A/Vic11, H3N2, IRR catalog No: FR-1041), A/Shanghai/1/2013 (A/SH13, H7N9, IRR catalog No: FR-1281), A/Hong Kong/33982/2009 (A/HK68, H9N2, IRR catalog No: FR-775) and pH1N1 viruses (H1N1, IRR catalog No: FR-187), or/and 50 ng/mL IL-15 (BD Pharmingen, San Diego, CA). Six days after incubation at 37°C 5% CO _2_, supernatant and B cells were collected for detection of anti-HA antibodies and ASCs, respectively.

### ELISpot assays

ELISpot assay of memory B cell IgG secretion was performed as previously described ^25^. Immobilon P membrane-based 96-well plates (Millipore, Billerica, MA) were coated overnight at 4°C with 10 *µ*g/mL H3N2 HA in phosphate-buffered saline (PBS) (40 *µ*l/well). PBS only was added to the negative-control wells. Plates were blocked with complete PRMI 1640 medium. Cells were plated at a density of 10 ^6^ per well in U-bottom plates and stimulated with CpG _2006_ ODN, six days after stimulation with A/Vic11 (H3N2, IRR catalog No: FR-1041). B cells were resuspended in complete medium containing either alkaline phosphatase-conjugated goat anti-human IgG (H-L) (KPL, Gaithersburg, MD) at 0.2 *µ*g/mL and incubated for 5h at 37°C in 5% CO _2_. The plates were washed, and then HA antibody secreting cell spots were developed with an alkaline phosphatase substrate kit (Vector Laboratories, Burlingame, CA). Spots were counted using a CTL ImmunoSpot plate reader and counting software (Cellular Technology Limited, Cleveland, OH).

### Statistical analysis

For multiple comparison of H3N2-specific IgG between-group (CpG _2006_ ODN alone or together with H3N2 or H7N9 viruses), one-way ANOVA was performed. Correlations between H3-specific antibodies and stalk-reactive antibodies, or A/Vic11-specific antibodies between plasma and CpG _2006_ ODN *±* memory B cells, were evaluated using the Pearson’s χ-squared test. Statistical analysis was performed using SPSS11.5 statistical software, as well as Prism 5 software. For all the statistical tests performed, p<0.05 was accepted as significant.

## Results

### Prevalence of H3-specific and stalk-reactive IgG

**Figure 1.**
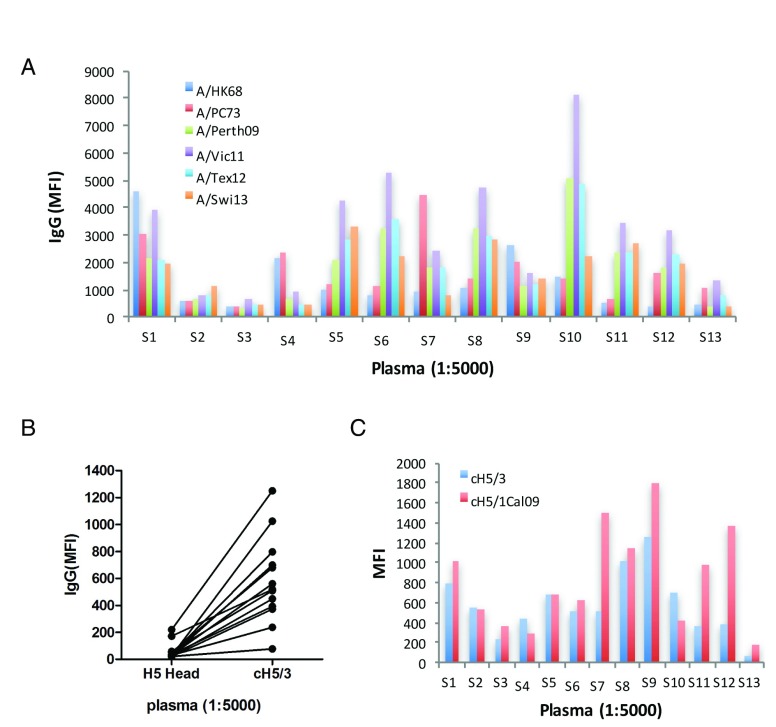
Anti-H3 stalk-reactive IgG antibodies in human plasma. Plasma was obtained from 13 donors, 12 of which (S1–S3, S5–S13) had been previously vaccinated within the past 5 years with trivalent or quadrivalent seasonal influenza vaccines. Plasma baseline anti-influenza IgG was measured by mPlex-Flu assay. ( **A**) Levels of IgG against distinct H3N2 strains. Each column depicts median fluorescence intensity (MFI), representing an individual donor. ( **B**) Levels of H3 stalk-reactive IgG. Each symbol and line represents one donor. ( **C**) IgG-binding to chimeric cH5/1 and cH5/3 proteins. This analysis was performed at a 1:5,000 dilution. Data can be found in Dataset 1 ^45^.

To evaluate the anti-influenza antibodies in plasma from 13 subjects and further infer influenza-specific memory B cells in their peripheral blood, we examined H3 HA-specific IgG levels using our mPlex-Flu assay. Six H3N2 strains, accommodating 45 years of antigenic drift of the swine origin influenza viruses (1968 to 2013), were selected to monitor the anti-H3 antibodies, including A/Hong Kong/1/1968 (A/HK68), A/Port Chalmers/1/1973 (A/PC73), A/Perth/16/2009 (A/Perth09), A/Victoria/361/2011(A/Vic11), A/Texas/50/2012 (A/Tex12) and A/Switzerland/9715293/2013 (A/Swi13). Antibodies against H3 strains were detected in all subjects. Of these, eight displayed high levels of H3-specific antibodies, while the other five had lower plasma baseline H3-reactive antibodies. Antibodies directed against A/Vic11 were high among all H3-specific IgG in 12 of the subjects. Only one subject displayed antibodies targeting the historical outbreak H3N2 strains, A/HK68 and A/PC73, which were higher than those against the more recent seasonal strains, A/Perth09, A/Vic11, A/Tex12 and A/Swi13 ( Figure 1). The emergence of H3N2-specific antibodies indicates that all subjects likely had prior exposure to H3 influenza viral antigen.

In order to determine the degree of H3 stalk-reactive antibodies induced by inactivated A/Vic11 viruses, cH5/3, a soluble HA construction that contains the stalk of H3 virus and the head of H5 virus, was used in our mPlex-flu assay. This recombinant HA protein allows for the direct detection of stalk-reactive IgG antibodies in polyclonal sera or plasma. As shown in Figure 1B, H3 stalk-reactive IgG was detected in plasma (dilution of 1:5,000) from 11 donors, with 4 to 11-fold lower than H3 strain-specific antibodies. For 4 of them, MFI values were greater than 3,000. Since most donors had a history of receiving seasonal influenza vaccines, we also examined the H1N1 stalk-reactive antibodies. Consistent with H3 stalk results, H1 stalk-binding IgG was detected in all subjects ( Figure 1C).

### H3N2 clade cross-reactive IgG secretion stimulated by inactivated A/Vic11 virus and CPG _2006_

**Figure 2.**
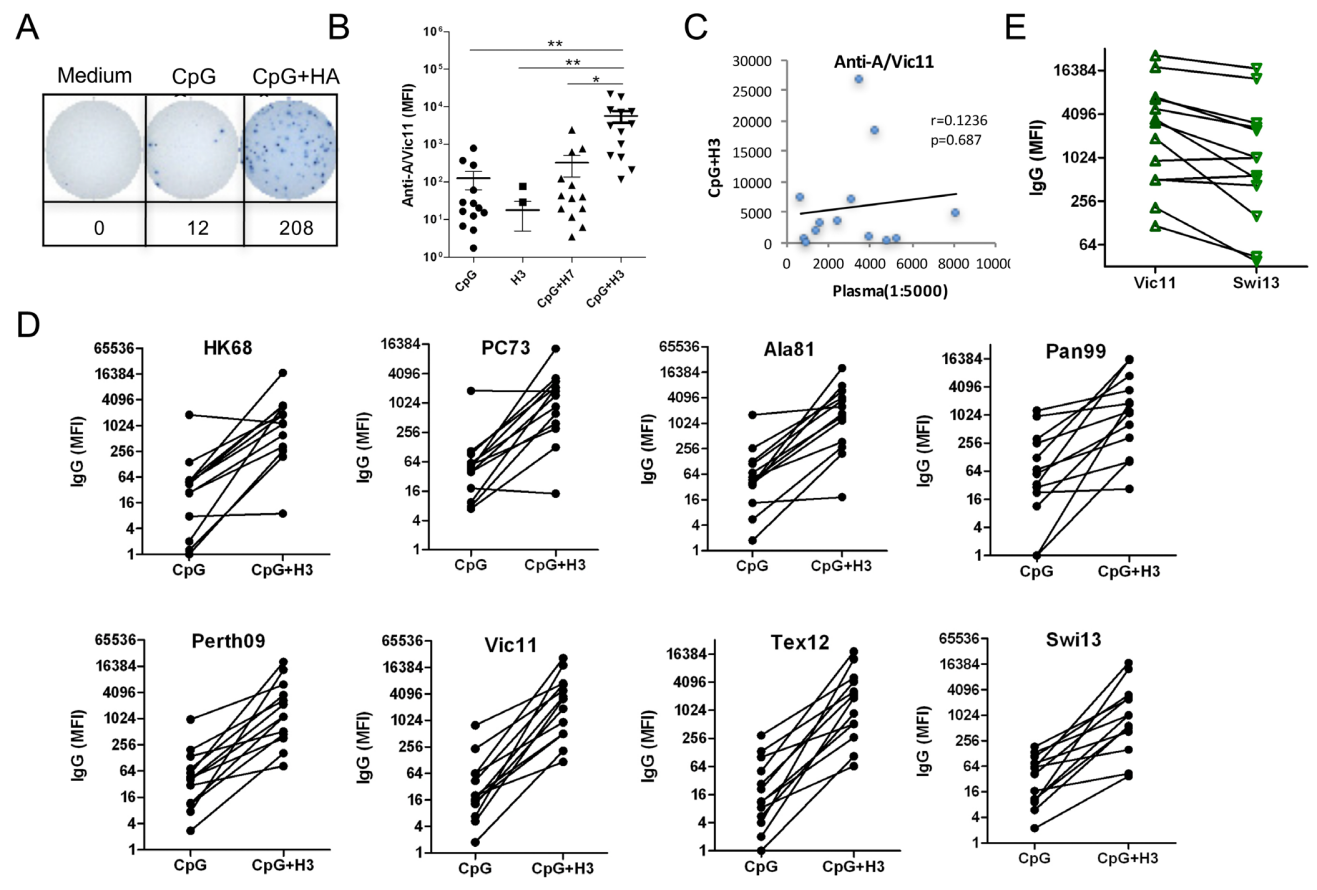
Secretion of H3 clade cross-reactive antibodies by B cells stimulated with inactivated A/Victoria/361/2011. Purified B cells were obtained by negative selection and stimulated with CpG _2006_ ODN alone or together with A/Vic11 (H3) or A/SH13 (H7) control BPL inactivated virus for 6 days *in vitro*. ( **A**) ASCs for H3 HA were examined by ELISpot assay. H3-binding antibodies in supernatants of activated B cells were monitored using beads bound with HA from H3N2 strains. ( **B**) A/Vic11-specific IgG. Each symbol represents an individual donor. One-way AVONA was used to evaluate the difference among different groups (* P<0.05; ** P<0.01). Results were verified by ELISpot after stimulation with A/Vic11. The values represent the number of anti-HA IgG specific antibody-secreting cells in 1.25 *x*10 ^5^ stimulated B cells/donor. ( **C**) Correlation between anti-A/Vic11 IgG levels in plasma and secretion of A/Vic11-specific IgG by activated B cells. ( **D**) Antibodies binding to H3 clades. ( **E**) Comparison of A/Vic11-specific IgG and A/Swi13-reactive IgG. Data from Dataset 2 ^46^.

To evaluate memory B cell response to H3N2 viruses, purified B cells were stimulated with CpG _2006_ ODN and BPL inactivated A/Vic11 virus. Six days after stimulation, ASCs for H3 HA from A/Vic11 were detected in both CpG _2006_ ODN with and without H3 virus (CpG-H3) groups. As shown in Figure 2A, the number of ASCs was greater in CpG-H3 group than in CpG alone group. H3N2 strain-specific antibodies in supernatants were assessed by mPlex-Flu assay. B cells from all donors displayed rapid antibody responses to inactivated A/Vic11 viruses. Stimulation with CpG _2006_ ODN and H3 viruses resulted in a significant increase in antigen-specific IgG production, compared with H3, CpG and CpG with A/Shanghai/1/2013 (A/SH13) (CpG-H7) groups ( Figure 2B). We also analyzed the relationship between the levels of A/Vic11-specific antibodies in plasma and IgG production by activated B cells. No correlation between antibody recall responses and plasma baseline IgG against A/Vic11 was detected (r=0.124, P=0.687) ( Figure 2C).

We next measured clade cross-reactive IgG induced by A/Vic11 viruses against selected recombinant HAs from seven heterovariant H3N2 strains spanning 45 years (1968–2013), A/HK68, A/PC73, A/Alabama/1/1981 (A/Ala81), A/Panama/1/2007/1999 (A/Pan99), A/Perth09, A/Tex12, and A/Swi13. Following stimulation with recent seasonal virus A/Vic11, activated B cells from all donors showed increased production of IgG targeting recent seasonal strains (A/Perth09, A/Vic11, A/Tex12 and A/Swi13), while increases in IgG against historical strains (A/HK68, A/PC73, A/Ala81 and A/Pan99) were detected in 11 donors (84.6%). One subject who had low baseline A/Vic11-specific antibodies showed much weaker antibody recall responses to A/Pan99, A/Perth09, A/Vic11, A/Tex12 and A/Swi13 strains than to the historical strains, A/HK68, A/PC73 and A/Ala81. In another subject, low levels of anti-A/HK68, A/PC73, A/Ala81 and A/Pan99, but high levels of anti-A/Perth09, A/Vic11, A/Tex12 and A/Swi13 were present after *in vitro* B cell stimulation ( Figure 2D). Interestingly, antibodies against the most recent seasonal strain A/Swi13 were lower than those against A/Vic11 ( Figure 2E).

### A/Vic11 stimulation induced cross-reactive IgG

We then tested the IgG induced by inactivated H3N2 viruses binding to recombinant HAs from influenza A strains, H1, H2, H5, H6, H7, H9, and B strains. For H1, six strains accommodating 89 years of antigenic drift of H1N1 influenza viruses from 1918 to 2009 were selected, including A/South Carolina/01/1918 (A/SC18), A/Puerto Rico/8/1934 (A/PR8), A/USSR/90/1977 (A/USSR77), A/Texas/36/1991 (A/Tex91), A/New Caledonia/20/1999 (A/NewCal99) and A/California/07/2009 (pdm A/Cali09). A/Japan/305/1957 (A/Jap57), A/Vietnam/1204/2004 (A/Viet04), A/Taiwan/2/2013 (A/TW13), A/TW13 and A/gf/HK99 represent H2, H5, H6 and H9 respectively, which are members of group 1. For group 2 influenza viruses, A/rhea/North Carolina/39482/1993 (A/rheaNC93), A/mallard/Netherlands/12/2000 (A/malNeth00) and A/SH13 represent three heterovariants of H7N9. B/Brisbane/60/2008 (B/Bris08), B/Mass/02/2012 (B/Mas12) and B/Phuket/2013 (B/Phu13) were selected for monitoring the cross reactivity to influenza B strains.

**Figure 3.**
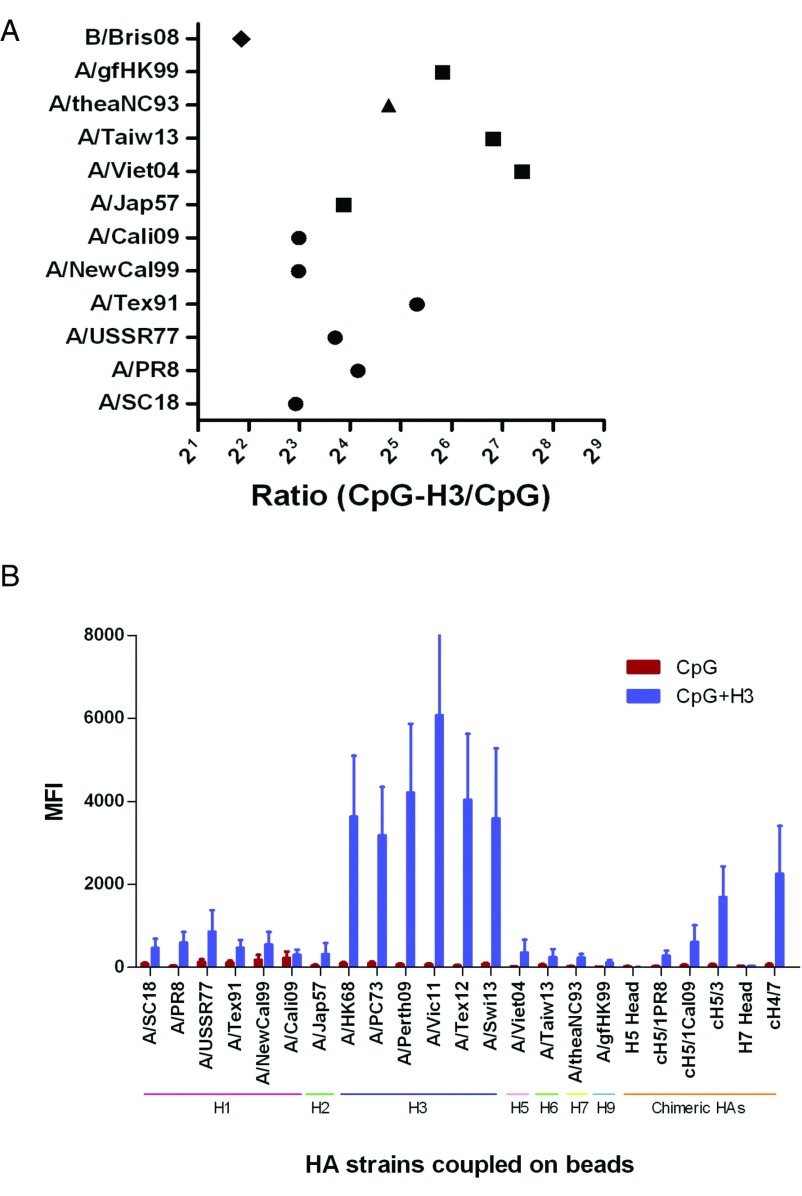
CpG _2006_ ODN + inactivated H3 virus boosts antigen-specific anti-H3 IgG antibody recall responses *in vitro*. B cells were obtained by negative selection, and then stimulated with CpG _2006_ ODN alone or together with A/Vic11 for 6 days. Cross-reactive antibodies binding to H1, H2, H5, H6, H7 and B influenza subtypes in B cell culture were measured by mPlex-Flu assay ^43^. ( **A**) Fold change in cross-reactive antibodies. All values of IgG levels (MFI) were subtracted from those of medium before calculating fold change. Only those values of IgG induced by CpG _2006_ ODN plus A/Vic11 viruses, which were greater than 100, were selected to calculate fold change. Each symbol represents the median of fold change in levels of IgG induced by CpG _2006_ ODN with H3 to IgG stimulated by CpG _2006_ ODN alone. ( **B**) CpG _2006_ ODN with H3 antigen induces a broad recall response to H3 influenza strains. Increases anti-HA IgG production to other non-H3 strains also occurred, but to a much lower extent. Data can be found in Dataset 3 ^47^.

As shown in Figure 3A, antibodies binding to group 1 and B strains, induced by the group 2 subtype, H3 viruses, were detected in supernatants derived from 8 and 4 donors, respectively, with increases of median of 3.6 to 166.4-fold. IgG in supernatants derived from 8 donors displayed broad cross-reactivity to HAs from 6 different H1N1 strains. Anti-A/Jap57 IgG was detected in supernatants of activated B cells from 3 donors, while anti-A/Viet04, anti-A/TW13 and anti-A/gfHK99 IgG were detected in B cells from 2 donors. For group 2, increases in levels of IgG against H7N9 strains, A/rheaNC93, were detected in 4 donors. For influenza B strains, B cells from 4 donors showed increases in yield of cross-reactive antibodies. Increases in anti-B/Bris08 IgG were shown in 3 donors, while two donors demonstrated an increase in anti-B/Phu13. Increases in anti-B/Wis10 and anti-B/Mass12 were not detected. As shown in Figure 3B, the largest response was enhancement of anti-H3 B cell recall responses, and a broad, but lower, increase in responses to other more molecularly distant strains also occurred. Notably, significant increases in IgG binding to chimeric HAs containing the H3, H1, and H7 HA stalk segments, but not the H7 or H5 head segments, were observed, strongly suggesting the presence of broadly-cross-reactive stalk antibodies targeting the conserved stalk regions ( Figure 3B).

### Stalk-reactive antibodies were induced by inactivated H3N2 A/Vic11 viruses

**Figure 4.**
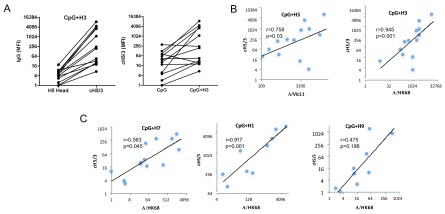
Induction of HA stalk-reactive antibodies by H3 viruses. B cells from healthy donors were stimulated with CpG _2006_ ODN alone or together with inactivated A/Vic11 (H3N2), A/SH13 (H7N9), A/Hong Kong/33982/2009 (A/HK09) (H9N2) or pdm A/Cali09 H1N1 viruses. The levels of IgG against H3 HA, H5 head and chimeric HA cH5/3 (H5 head and H3 stalk) are shown for individual subjects. ( **A**) Nine of 13 subjects displayed increases in stalk-reactive IgG after A/Vic11 (H3) stimulation. ( **B**, **C**) A correlation model assuming different coefficients for different anti-H3 strain-specific antibodies were fitted to evaluate the relationship between HA stalk-reactive and strain-specific IgG. Ordinate and abscissa units are mean fluorescence intensity (MFI). Data can be found in Dataset 4 ^48^.

H3 stalk-reactive antibodies were detected in activated B cells from 7 donors (53.8% of 13) after A/Vic11 stimulation ( Figure 4A). IgG against historical outbreak strains (e.g. A/HK68, A/PC7), which have divergent head domains but conserved stalk domains with the recent seasonal H3N2 strains, was detected in most donors (12 of 13). Therefore, we analyzed the relationship between stalk-reactive and A/HK68 or A/Vic11 strain-specific antibodies. There was a strong correlation between cross-reactive stalk antibodies and A/HK68 strain-specific IgG production (r=0.945, P<0.001). Interestingly, anti-A/Vic11 displays a significant but weaker correlation with cH5/3 stalk antibodies (r=0.758, p=0.03) than those against A/HK68( Figure 4B). To further investigate the reactivity of cross-reactive stalk antibodies induced by influenza A subtype strains to H3 stalk, B cells from were stimulated with HA proteins from A/SH13 (H7; group 2) and pandemic A/California/07/2009 (pdm A/Cali09, H1; group 1) and A/Hong Kong/33982/2009 (A/HK09) (H9; group 1). Positive correlations between anti-cH5/3 and anti-HK68 IgG induced by A/SH13 (r=0.563, p=0.045) and pdm A/Cali09 (r=0.917, p=0.001) were detected ( Figure 4C).

### IL-15 enhanced cross-reactive IgG secretion to H2N2 A/Victoria/361/2011 + CpG _2006_ stimulation

**Figure 5.**
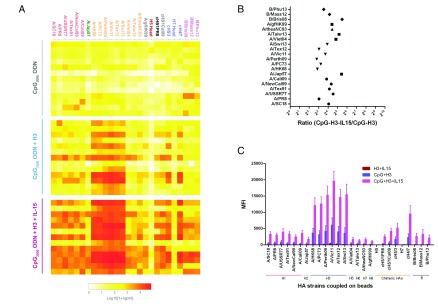
IL-15 increases cross-reactive antibody responses to H3N2 viruses. ( **A**) B cells from healthy donors were co-stimulated with CpG _2006_ ODN, inactivated A/Vic11 viruses and IL-15. Strain-specific and HA stalk-reactive IgG in supernatants of activated B cells were detected after 6 days. ( **B**) Fold change in cross-reactive antibodies. ( **C**) Correlation between influenza specific antibodies and HA antigenic sequence. IL-15 increased the concentration of anti-HA reactive IgG, but does not alter the distribution of cross-strain specificity. Data can be found in Dataset 5 ^49^.

To assess if IL-15 has an influence on B cell recall responses to H3N2 viruses, we added IL-15 to cell cultures with CpG _2006_ ODN and BPL inactivated A/Vic11 influenza virus, or with CpG _2006_ ODN alone. The supernatants at day 6 were measured for IgG production against influenza A strains, including group 1 and group 2, B strains and chimeric HA proteins by mPlex-Flu assay. Fold change values were calculated as described in Figure 3. As shown in Figure 5A, cross-reactive antibody responses to the specific A/Vic11 viruses were enhanced in cell culture derived from all donors upon IL-15 stimulation.

First we analyzed the influence of exogenous IL-15 on the *in vitro* B cell recall production of A/Vic11-specific IgG from all subjects. All subjects showed increased B cell secretion of A/Vic11 HA-specific antibodies after IL-15 treatment (median 13.1). Increases in cross-reactive antibodies binding to H3N2 heterovariant, A/HK68, A/PC73, A/Perth09, A/Tex12 and A/Swi13 were detected in all subjects, with median of 7.3, 9.4, 6.9, 8.2, 15, respectively. For influenza A subtype strains, IL-15 showed strong upregulation, with fold change in IgG against A/SC18 (median 16.6), A/PR8 (median 8.2), A/USSR77 (median 23), A/Tex91 (17.5), A/NewCal99 (20.3) and pdm A/Cali09 (14.3), A/Jap57 (42.6), A/Viet04 (28.3), A/TW13 (35.6), A/rheaNC93 (21.4) and A/gfHK99 (21.1). Ten donors displayed increases in IgG against B subtypes, B/Bris08 (median 44.6), B/Mass12 (17.8) and B/Phu13 (11.6) ( Figure 5B). We next analyzed the relationship between strain-reactive IgG and HA stalk types, which revealed increases in stalk-reactive IgG against H1, H3, and H7 stalk regions that increased greatly with IL-15 + CpG _2006_ ODN stimulation *in vitro*. ( Figure 5C).

### IL-15 increased secretion of stalk-reactive IgG

**Figure 6.**
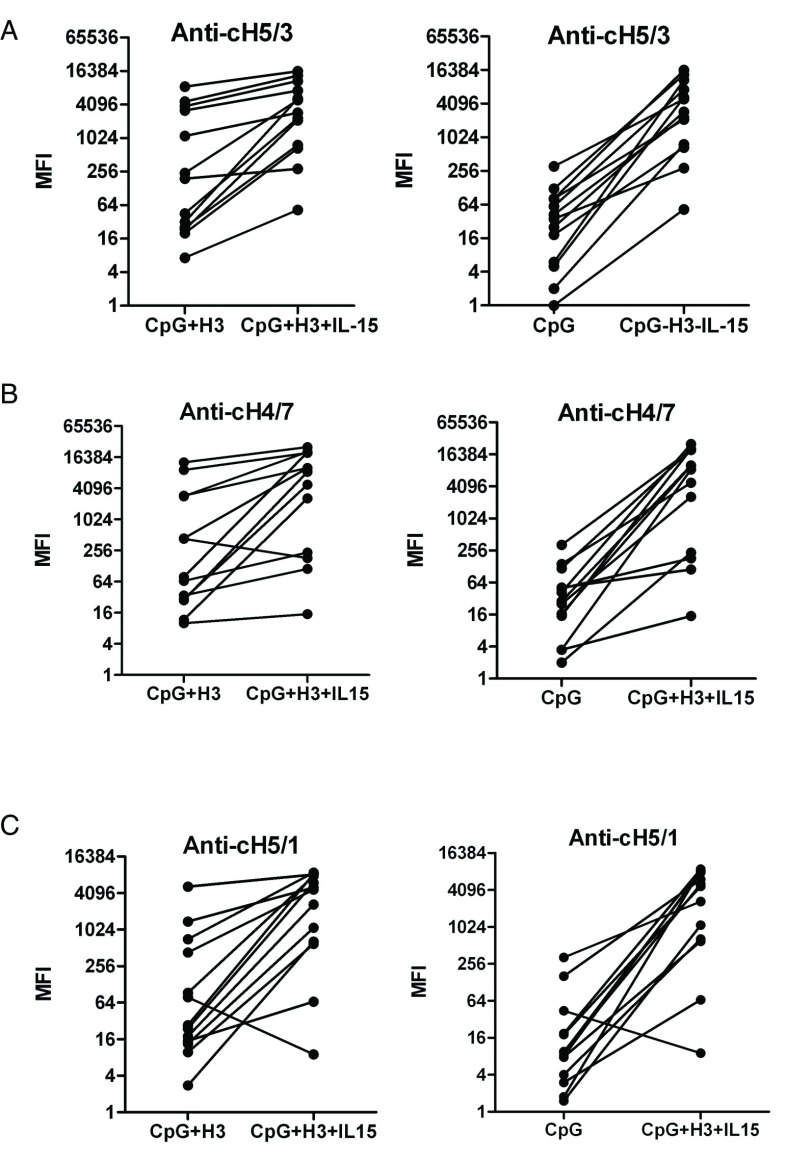
Stalk-reactive antibody responses to H3 viruses are enhanced by IL-15. Purified B cells were costimulated with CpG _2006_ ODN, A/Victoria/361/2011 viruses and IL-15. Stalk-reactive IgG in supernatants was detected at day 6. Chimeric molecules cH5/3, cH4/7 and cH5/1 were used to measure antibodies against the H3, H7, and H1 stalks, respectively. Each symbol and line represents an individual donor. ( **A**) H3 stalk-reactive antibodies. ( **B**) H7 Stalk-reactive antibodies. ( **C**) H1 stalk-reactive antibodies. Data can be found in Dataset 6 ^50^.

Although stalk-reactive antibodies induced by seasonal H3N2 viruses were detected in this study, these antibodies were lower than those against entire H3 HA. To assess whether H3 stalk-reactive antibodies can be enhanced by IL-15, we measured IgG binding to cH5/3. As shown in Figure 6A, anti-cH5/3 IgG increased after costimulation with CpG _2006_ ODN, H3 viruses and IL-15, compared with CpG-H3 and CpG groups. Since most influenza subtypes, either influenza A, including group 1 and group 2 subtypes, or influenza B strains, share conserved stalk epitopes, we also evaluated IgG against H7 and H1 stalk using cH5/1 (containing H5 head and H1 stalk) and cH4/7 (containing H4 head and H7 stalk). Anti-cH5/1( Figure 6B) and anti-cH4/7 ( Figure 6C) IgG were higher in CpG-H3-IL-15 groups than in CpG-H3 and CpG alone groups. H7 and H1 stalk-reactive antibodies were upregulated along with H3 stalk-reactive antibodies.

## Discussion

The majority of adults possess pre-existing IgG antibodies against influenza viruses from prior infection and/or vaccination ^51^, primarily directed against the immunodominant globular head domain of the HAs. Antibodies against the conserved, immuno-subdominant HA stalk domain are generally detectable at very low levels, if at all, despite being broadly protective against multiple influenza strains and subtypes ^52^. In this study, we analyzed strain-specific and H3 stalk-reactive antibodies in plasma from donors, and found high levels of anti-H3 specific IgG in all donors. Of these, 12 subjects had high IgG levels against the recent seasonal H3N2 strain A/Vic11, likely by vaccination or infection. One donor, who had never received a seasonal influenza vaccine, showed much lower IgG binding to A/Vic11, but higher anti-A/HK68 IgG levels. Interestingly, 11 of 13 subjects had detectable H3 stalk-reactive IgG at levels only 4–11 fold lower than H3 strain-specific IgG antibodies. The presence of pre-existing stalk-reactive antibodies suggests that stalk-specific memory B cells exist in memory B cell pool, a hypothesis supported by the results from the *in vitro* B cell stimulation ELISpot experiments.

Immunological memory against influenza following immunization is the corner-stone for prophylactic vaccination programs ^53^. Most adults possess a low (0.1–1.0% of total IgG memory B cells) but consistent base line of influenza virus-specific memory B cells ^25^. Stalk-reactive memory B cells have generally been reported at very low frequencies, suggesting minimal effective protection ^16, 20, 54^. Interestingly, broad cross-reactive antibodies have been detected in participants who received the pdm A/Cali09 vaccine ^24^, which is distinct from the prior seasonal H1N1 strain. Competition of memory B cells responding to either common or rare antigens is hypothesized to regulate the appearance of cross-reactive IgG against influenza HA ^20, 24^. Repeat exposure to common influenza strains primarily boosts head-reactive responses and limits the expansion of B cells secreting broadly neutralizing antibodies. In contrast, repeated exposure to diverse influenza strains boosts antibodies against highly conserved influenza HA regions, such as the the stalk ^24^ or a conserved Ca ^2+^-binding region ^55^ on the globular head.

In this study, we focused on enhancing the memory B cell recall response to H3N2 subtype influenza viruses following H3 stimulation *in vitro* in the presence of CpG _2006_ ODN ± IL-15. Class switched IgG antibodies against a panel of H3 strains spanning 45 years from 1968 to 2013 were analyzed. Increases in antibodies against recent seasonal strains were present in all subjects, while induction of IgG antibodies binding to historical H3 strains (prior to the birth year of the subjects) only occurred in 11 subjects. This is consistent with the prevailing hypothesis that antigenic drift leads to low vaccine efficacy ^5– 7, 10^. Decreases in responses to Anti-A/Swi13 also explain the low vaccine efficacy in 2014–2015 influenza season. Moreover, we found that antibodies induced by H3 viruses, especially in the presence of IL-15, broadly bound to group 1 HAs, with moderate reactivity against group 2 and B influenza subtypes. These results are consistent with findings by other groups of cross-reactive IgG that react against both H1 and H3 influenza strains, primarily against epitopes on the HA stalk regions ^19, 21^.

CpG _2006_ ODN stimulation has been shown to drive *in vitro* differentiation of both CD27 ^−^ and CD27 ^+^ human B cells to the plasma cell phenotype ^29, 44^. Antibody production rates after CpG _2006_ ODN stimulation appear to be modulated by IL2R *γ* signaling cytokines ^29^. *In vivo*, mouse vaccine studies have noted that CpG adjuvanted influenza vaccination increases anti-HA IgG titers in young, but not older mice ^56^. Our results suggest that the stimulation of B cells *in vitro* with inactivated H3 influenza in combination with CpG _2006_ ODN and IL-15 not only stimulate increased anti-HA IgG, but appears to increase levels of secreted cross-reactive IgG compared with CpG alone. IL-15 has been reported to overcome immundominance of antigens in CD8 T cell activation ^57^. Further work will need to be done to determine if the addition of IL-15 to CpG adjuvanted influenza vaccines would boost protective anti-HA IgG production *in vivo* in older individuals.

Prior characterization of broadly neutralizing IgG antibodies by other groups has demonstrated that cross-activity results from stalk-reactive antibodies ^26, 58^. Using the mPlex-Flu assay, we found that moderate to high levels of H3 stalk-reactive antibodies could be induced *in vitro* after CpG _2006_ ODN stimulation of memory B cells from 8 subjects (53.3%). These H3 stalk-reactive antibodies emerged along with IgG that bound the stalk regions of H1 and H7 subtypes, which share conserved epitopes with H3 viruses. Using a liner correlation model, we found that levels of antibodies binding to A/HK68 were positively related to H3 stalk-reactive antibodies, suggesting that clade cross-reactivity was likely due to the conserved epitopes in H3 stalk. This correlation also strongly existed between IgG antibodies binding to the H3 stalk and anti-A/HK68 antibodies responding to inactivated H7N9 viruses, suggesting that H3 stalk-specific memory B cells responded to H7N9 subtypes. Interestingly, stalk-specific IgG recall responses were not seen in memory B cells from the infected/un-vaccinated participant (S4), although they had detectable IgG antibodies against historical outbreak H3 strains after *in vitro* CpG _2006_ ODN + IL-15 stimulation.

Although the production of stalk-reactive antibodies indicated activation of stalk-specific memory B cells, we found both strain cross-reactive and stalk-reactive IgG antibodies present at much lower levels than H3 strain-specific anti-HA IgG antibodies. Given the desirability of inducing broadly cross-reactive anti-HA stalk-reactive IgG antibodies, vaccination strategies to achieve this goal need to be developed. In this study, we evaluated the effect of supplemental IL-15 on B cell recall responses to inactivated A/Vic11 viruses. We demonstrated that the B cell recall responses to H3 viruses were enhanced by CpG _2006_ ODN + IL-15, with increases of 6.9 to 15-fold in IgG production. Broadly cross-reactive IgG antibodies were observed to bind to group 1, group 2 and B strain influenza HA subtypes following CpG _2006_ ODN + IL-15 stimulation, with median of increase of 22.3-fold for group 1, 21.4-fold for both group 2 and 17.6-fold for B subtypes compared to CpG _2006_ ODN stimulation alone. This demonstrated IL-15 greatly augmented recall secretion of broadly cross-reactive anti-influenza HA IgG antibodies. Not surprisingly, there was a negative correlation between antigenic sequence dissimilarity of the stimulating influenza strain and recall IgG antibody levels.

## Conclusions

In conclusion, broadly cross-reactive anti-influenza stalk-binding IgG antibodies exist in individuals exposed to influenza strains. Seasonal H3N2 virus exposure, through vaccination or infection, can induce memory B cells that bind to the conserved stalk region of HAs. *In vitro* recall responses to these stalk-reactive antibodies can be enhanced by IL-15. These results suggest the potential for IL-15 augmentation of adjuvant to overcome immunodominance of influenza HA head region epitopes as a potential vaccine boosting strategy to increase levels of broadly cross-reactive anti-influenza HA IgG antibodies.

## Data availability

Figshare: *Dataset 1*: Anti-H3 stalk reactive antibodies in human plasma. https://doi.org/10.6084/m9.figshare.5481565.v2 ^45^

Figshare: *Dataset 2*: Secretion of H3 clade cross-reactive antibodies by B cells stimulated with inactivated A/Vic11. https://doi.org/10.6084/m9.figshare.5498080.v1 ^46^

Figshare: *Dataset 3*: Influenza viruses induce cross-reactive antibody responses *in vitro*. https://doi.org/10.6084/m9.figshare.5498071.v2 ^47^

Figshare: *Dataset 4*: Induction of HA stalk-reactive antibodies by H3 viruses. https://doi.org/10.6084/m9.figshare.5498116.v1 ^48^

Figshare: *Dataset 5*: IL-15 increases cross-reactive antibody responses to H3N2 viruses. https://doi.org/10.6084/m9.figshare.5498152.v1 ^49^

Figshare: *Dataset 6*: Stalk-reactive antibody responses to H3 viruses enhanced by Il-15. https://doi.org/10.6084/m9.figshare.5498197.v1 ^50^

All data are available under the terms of the Creative Commons Attribution 4.0 International license (CC BY 4.0).
