# Supplementary material for: IL-15 enhances cross-reactive antibody recall responses to seasonal H3 influenza viruses in vitro
Source: F1000Res. 2017 Nov 15;6:2015. [Version 1] doi: 10.12688/f1000research.12999.1 (PMC5801566; doi:10.12688/f1000research.12999.1)
Supplement: Peer review history

# Peer review: Referee response for version 1

**DOI:** 10.5256/f1000research.14095.r29326

**Published:** 2018-02-02

**Authors:** Raghavan Varadarajan, Poorigali R. Sowmya

**Affiliations:** 1 Molecular Biophysics Unit, Indian Institute of Science, Bangalore, India; 2 Indian Institute of Science, Bangalore, India

**Reviewer comments:**

 In the current study, authors examined the human memory B cell IgG recall responses to H3N2 influenza virus in the presence of CpG _2006_ ODN activation with IL-15 co-stimulation *in vitro*. They demonstrated that stalk reactive IgG antibodies induced by B cell exposure to H3 viruses *in vitro*, in the presence of CpG2006 ODN, are enhanced by IL-15 co-administration. In addition, IgG antibodies elicited by H3 viruses and/or IL-15 broadly bound to influenza HAs from both group 1 and group 2 influenza strains, which suggests potential use of CpG adjuvants and/or IL-15 agonists in influenza vaccination strategies. The use of IL-15 to enhance responses to immunization has been studied previously (see for example, PMID:24706798 which the authors could cite). However in that study, it was T-cell rather than antibody responses that were found to be protective. In this context, it would be important in a future study to examine whether the induced, cross-reactive antibodies show any protective activity in an appropriate assay. Overall, the manuscript is clearly written and interesting to read. However, the authors could consider the following additional points to improve clarity.

1. Please include list of abbreviations used in the manuscript.
2. Page No. 4.
  What are the concentration of whole HA or the head segments of influenza group 1, group 2, B strain and chimeric HA used for the mplex-Flu assay?
  What is the concentration of PE conjugated goat anti-human IgG (mplex-Flu assay) used in the current study?
3. Page No. 5.
  On what basis was the concentration of IL-15 (50 ng/mL) fixed for the study? Did the authors check effects of different concentrations? If yes, what was the effect?
  (It is important to screen the IL-15 concentration because, in a previous NHP study antibody responses were sensitive to IL-15 dose with a slight increase at lower doses, but a decrease at higher doses ( Yin et al., High dose of plasmid IL-15 inhibits immune responses in an influenza non-human primates immunogenicity model. Virology; 393(1), 2009, 49-55.).
4. In the result section, authors mention “As shown in Figure 1B, H3 stalk-reactive IgG was detected in plasma (dilution of 1:5,000) from 11 donors, with 4 to 11-fold lower than H3 strain-specific antibodies. For 4 of them, MFI values were greater than 3,000”. However,  the maximum MFI values given in data set is 1250. Also there is no caption for Figure 1C.a
5. Page No. 9. H2N2 should be changed to H3N2.
6. Use A/Vic11 abbreviation consistently throughout the manuscript.
7. References should be uniform.

We have read this submission. We believe that we have an appropriate level of expertise to confirm that it is of an acceptable scientific standard.

# Peer review: Referee response for version 1

**DOI:** 10.5256/f1000research.14095.r28740

**Published:** 2018-01-08

**Authors:** Qibo Zhang

**Affiliations:** 1 Department of Clinical Infection, Microbiology and Immunology, University of Liverpool, Liverpool, UK

The authors studied anti-influenza HA IgG antibody levels using a multiplex assay in adults who exposed to H3 viruses previously and/or received influenza vaccination and demonstrated that stalk-reactive antibody recall responses induced by seasonal H3 viruses and CpG ODN can be enhanced by IL-15. The manuscript is generally well written and  clearly presented. It may be helpful that authors could add a bit more discussion as to how IL-15 with/without CpG activate the B cell recall response leading to the anti-stalk antibody production.

 Minor point: page 9, top right

 "B cells from ?? were stimulated with HA proteins from A/SH13"

I have read this submission. I believe that I have an appropriate level of expertise to confirm that it is of an acceptable scientific standard.
